# Cognitive Impairment in Multiple Sclerosis Is Reflected by Increased Susceptibility to the Sound-Induced Flash Illusion

**DOI:** 10.3389/fneur.2019.00373

**Published:** 2019-04-12

**Authors:** Yavor Yalachkov, Heinrich Johannes Bergmann, Dilara Soydaş, Christian Buschenlange, Laura Yasmine Fadai Motlagh, Marcus J. Naumer, Jochen Kaiser, Stefan Frisch, Marion Behrens, Christian Foerch, Johannes Gehrig

**Affiliations:** ^1^Department of Neurology, University Hospital Frankfurt, Frankfurt am Main, Germany; ^2^Institute of Medical Psychology, Goethe-University, Frankfurt am Main, Germany; ^3^Institute of Psychology, Goethe-University, Frankfurt am Main, Germany

**Keywords:** multiple sclerosis, cognitive deficits, screening test, sound-induced flash illusion, neuropsychological impairment

## Abstract

**Objective:** To determine whether the performance of multiple sclerosis (MS) patients in the sound-induced flash illusion (SiFi), a multisensory perceptual illusion, would reflect their cognitive impairment.

**Methods:** We performed the SiFi task as well as an extensive neuropsychological testing in 95 subjects [39 patients with relapse-remitting MS (RRMS), 16 subjects with progressive multiple sclerosis (PMS) and 40 healthy control subjects (HC)].

**Results:** MS patients reported more frequently the multisensory SiFi than HC. In contrast, there were no group differences in the control conditions. Essentially, patients with progressive type of MS continued to perceive the illusion at stimulus onset asynchronies (SOA) that were more than three times longer than the SOA at which the illusion was already disrupted for healthy controls. Furthermore, MS patients' degree of cognitive impairment measured with a broad neuropsychological battery encompassing tests for memory, attention, executive functions, and fluency was predicted by their performance in the SiFi task for the longest SOA of 500 ms.

**Conclusions:** These findings support the notion that MS patients exhibit an altered multisensory perception in the SiFi task and that their susceptibility to the perceptual illusion is negatively correlated with their neuropsychological test performance. Since MS lesions affect white matter tracts and cortical regions which seem to be involved in the transfer and processing of both crossmodal and cognitive information, this might be one possible explanation for our findings. SiFi might be considered as a brief, non-expensive, language- and education-independent screening test for cognitive deficits in MS patients.

## Introduction

Depending on the disease course, employed methods and cut-off-scores, prevalence rates of cognitive impairment in multiple sclerosis (MS) vary between 40 and 70% (1, 2). Due to financial, personnel and time limitations in the clinical routine, sometimes mild cognitive deficits in MS are not subjected to a further diagnostic investigation. An inexpensive, easy to apply language-independent screening tool for global cognitive deficits would facilitate the neuropsychological diagnostic process in MS.

The typical profile of neuropsychological impairment in MS encompasses slowed cognitive processing speed, episodic memory deficits, executive dysfunction, impaired verbal fluency, and visuospatial perception (3). Neuroimaging as well as neuropathological findings suggest that cortical regions as well as white matter tracts and deep gray matter areas which are involved in cognitive processing are often affected by MS (4). Interestingly, similar anatomical structures and functional circuits have been implied in the integration of perceptual information from different sensory channels, too (5–8). Therefore, lesions in these areas should result not only in neuropsychological impairment but also in an altered multisensory perception. In this study, we examined the principal utility of a multisensory perceptual task, the sound-induced flash illusion (SiFi) (9–11), as a marker for global cognitive impairment in MS.

**Figure 1:**
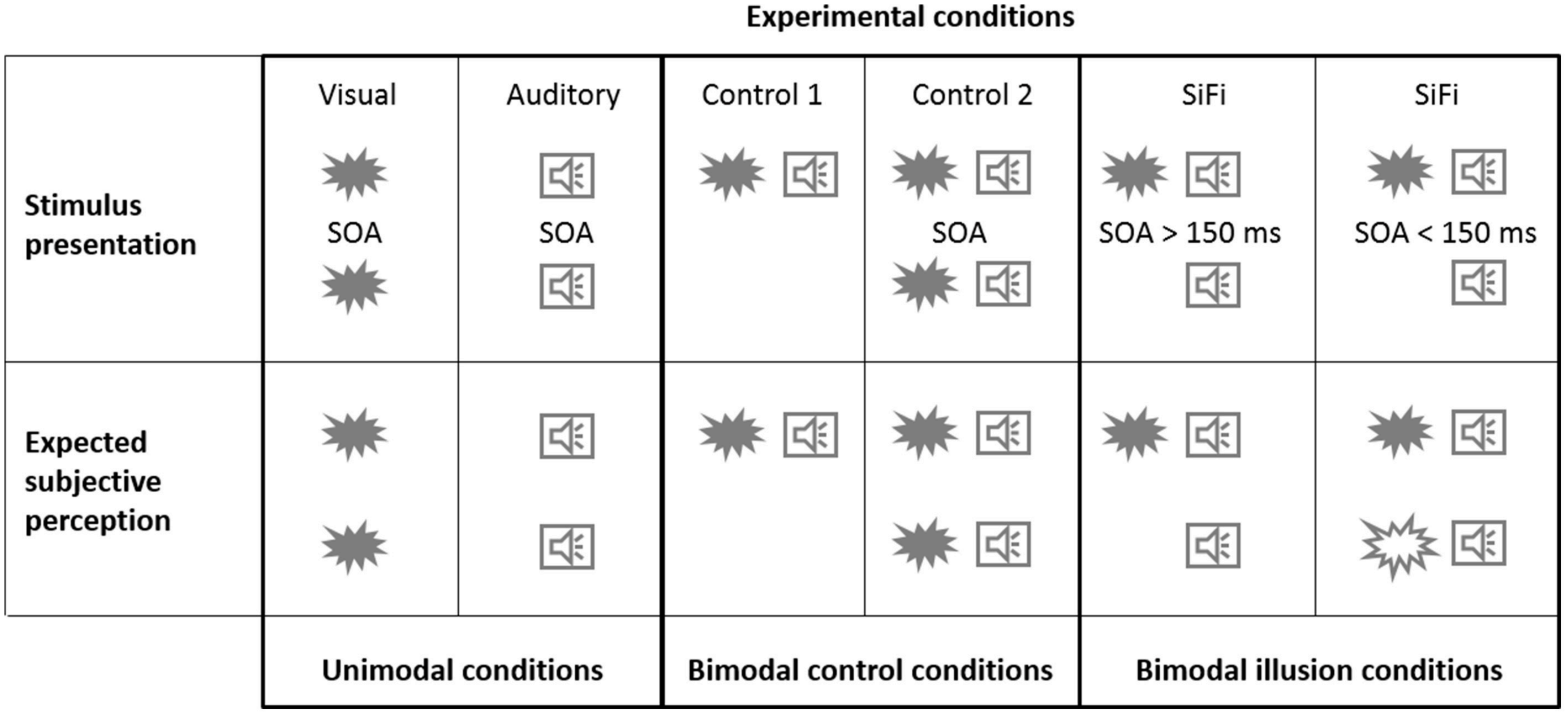
Stimulus presentation. Experimental stimuli (illustrated here by a flash or a beep symbol) were presented either in only one (visual/auditory unimodal conditions) or in both of the sensory modalities (control/illusion bimodal conditions). The stimulus onset asynchrony (SOA) of two subsequent stimuli could amount to 0 ms (only one stimulus), 50 ms, 100 ms, 150 ms, 200 ms, 250 ms, 300 ms, or 500 ms. *Expected subjective perception*. In the illusion condition, if shortly after the first stimuli a second auditory beep is presented, some of the subjects perceive two instead of one visual flash, e.g., the inputs from the two different sensory modalities fuse and the subjects perceive a second, non-existing visual flash. Healthy subjects usually report perceiving this illusion if the time interval between the beeps is shorter than 150 ms. For interstimulus intervals longer than 150 ms the illusion is less frequently or not perceived at all by healthy subjects.

The SiFi is a multisensory illusion, where two auditory beeps are presented to the subject with a short interval between them (12). A single visual flash is presented together with the first auditory beep. If the time interval between the two auditory beeps is less than 150 ms, healthy subjects perceive two instead of one visual flash, e.g., the inputs from the two different sensory modalities fuse and the subjects perceive a second, non-existing visual flash (Figure 1, bimodal illusion conditions) (9, 12). For interstimulus intervals longer than 150 ms the illusion is less frequently or not perceived at all. However, some patient groups seem to exhibit different perceptual patterns. For example, people with mild cognitive impairment (MCI) report seeing the second visual flash even at longer interstimulus intervals (up to 300 ms) (13). Similarly, older adults with a history of falling continue experiencing the illusion for time intervals of up to 270 ms (14).

Since both perception and cognitive processes depend strongly on viable brain connectivity, we hypothesized that neuronal damage as seen in MS would be associated not only with dysfunctional multisensory perceptual processes (and thus with increased susceptibility to the illusion) but also with cognitive impairment in MS patients. Hence, MS patients perceiving the illusion at interstimulus intervals long enough to disrupt the illusion for healthy subjects (i.e., > 150 ms) should exhibit inferior performance on neuropsychological testing.

## Materials and Methods

### Study Population

Fifty-nine multiple sclerosis (MS) subjects (43 relapse-remitting (RRMS) and 16 primary or secondary progredient (PMS) patients) and forty-one healthy controls (HC) were included initially. Five of them (four RRMS and one HC) did not complete the full experiment because they discontinued the task or did not show up for the second measurement. The data of the remaining 55 MS patients (39 RRMS and 16 PMS) and 40 HC were included in the analysis. Patients were recruited via the neurology department at the University Hospital in Frankfurt am Main, Germany. The diagnosis of MS was established according to the 2010 diagnostic criteria for MS (15). This study was carried out in accordance with the recommendations of the ethics committee of the University of Frankfurt Medical Faculty with written informed consent from all subjects. All subjects gave written informed consent in accordance with the Declaration of Helsinki. The protocol was approved by the ethics committee of the University of Frankfurt Medical Faculty.

The average age of the MS group (40 female, 15 male) was 43.1 years (SD 13.7). The RRMS subgroup consisted of 29 female and 10 male and the PMS subgroup of 11 female and 5 male subjects (mean age of RRMS patients 38.1 years [SD 11.3], mean age of PMS patients 55.4 years [SD 10.99]). The average visual acuity of the RRMS group was 0.81 for the left as well as the right eye (SD left 0.18, SD right 0.17). The average visual acuity of the PMS group was 0.70 for the left and 0.71 for the right eye (SD left 0.16, SD right 0.21). The mean number of years of education (YoE) was 13.2 (SD 0.9). The mean number of YOE for the RRMS patients was 13.3 (SD 0.8) and for the PMS 13.1 (SD 0.99). The average age of the HC group (30 female, 10 male) was 41.5 years (SD 14.7). The average visual acuity of the HC group was 0.86 for the left and 0.83 for the right eye (SD left 0.14, SD right 0.20). The mean number of years of education was 13.8 (SD 0.5). We ensured that no subject had a severe hearing loss. More detailed information on the basic demographic information can be found in Table 1 and in Results.

**Table 1:** Main clinical characteristics for healthy controls (HC), relapse-remitting multiple sclerosis (RRMS), and progredient multiple sclerosis (PMS) patients.

**Variable**	**HC mean (30 f, 10 m)**	**HC std. dev**.	**RRMS mean (29 f, 10 m)**	**RRMS std. dev**.	**PMS mean (11 f, 5 m)**	**PMS std. dev**.
Age	41.45	14.69	38.10	11.34	55.44	10.99
Years of education	13.83	0.50	13.26	0.83	13.06	1.00
Vision left eye	0.87	0.14	0.79	0.18	0.72	0.19
Vision right eye	0.84	0.20	0.81	0.16	0.74	0.21
Disease duration (years)			6.81	7.39	13.21	12.70
EDSS			2.41	1.61	4.38	1.23
VAS relative score	−0.03	0.21	−0.17	0.33	−0.02	0.78
RCFT_IR (raw score)	22.60	5.44	17.71	7.94	14.78	7.48
SDMT (raw score)	54.65	10.61	46.00	12.14	33.13	9.68
VLMT total (raw score)	60.33	7.97	54.21	8.59	50.06	9.10
VLMT 5–7 (raw score)	0.60	1.57	1.10	2.17	0.94	1.84
PASAT (raw score)	9.41	6.73	16.56	11.22	26.00	16.77
TMT-A (raw score)	26.73	8.61	37.72	15.20	46.88	18.07
TMT B/A (raw score)	2.27	0.67	2.15	0.92	2.39	0.65
RWTp (raw score)	26.25	6.25	20.36	6.38	18.75	6.47
RWTs (raw score)	39.60	9.40	31.49	9.14	31.56	10.68
WST (raw score)	34.13	2.67	28.90	7.07	29.38	8.94
BDI	3.78	4.44	8.92	7.27	14.73	9.97
WST (z-score)	0.78	0.55	0.11	0.68	0.29	0.71

### Design and Data Collection

The study comprised two sessions which were conducted on two different days to reduce the effect of fatigue on performance. Demographics and basic sensory functions (visual acuity, hearing ability) were recorded during the first session. Visual function was measured using a Snellen eye chart. Auditory function was measured using a hearing test with an audiogram output (https://hearingtest.online/). The test files are based on the ISO 389-7:2005 international standard and use third octave band warble tones in order to minimize room and headphone resonance. We employed Sennheiser HD 201 headphones. Half of the participants was scheduled to complete a neuropsychological test battery during the first session consisting of pre-task Visual Analog Scale (VAS 1) on subjective performance capability, Rey Complex Figure Test (RCFT) (16), Symbol Digit Modalities Test (SDMT) (17), Verbaler Lern- und Merkfähigkeitstest (VLMT, a German adaptation of the Rey Auditory Verbal Learning Test) (18), Paced Auditory Serial Addition Test (PASAT) (19), Trail Making Test (TMT) (20), Regensburger Wortflüssigkeits-Test (RWT) (21), Wortschatztest (WST, a German vocabulary test) (22), Beck Depression Inventory (BDI) (23) and post-task Visual Analog Scale (VAS 2) on subjective performance capability to evaluate changes in fatigue related to the neuropsychological testing. During the second session, which was scheduled not more than 6 weeks after the first one, participants completed the sound-induced flash illusion task (SiFi). The other half of the participants completed the tasks in reversed sequence (day 1: SiFi, day 2: neuropsychology).

For the SiFi, we employed a well-established experimental design already used in studying multisensory perceptual patterns in patients with mild cognitive impairment (13). The visual stimuli were presented on a 15.6″ laptop. The visual stimulus consisted of a white circular disk, subtending approximately 2° of visual angle. This disk was placed 8° of visual angle below the fixation cross. The presentation duration of the disk was 16 ms. The auditory stimulus consisted of a 16 ms, 3,500 Hz pure tone with a total rise- and decay-time of 20 μs at a sound pressure level of 68 dB(A). Auditory stimuli were presented using closed, circum-aural headphones (Sennheiser HD 201) to reduce any ambient noise.

We used a repeated measures design. Each trial began with a fixation cross presented at the center of screen. Participants were instructed to maintain fixation on the cross throughout the measurements. Experimental stimuli were presented either in only one or in both of the sensory modalities [factor “modality” with levels “visual” (V), “auditory” (A), and “audio-visual” (AV)]. In the V-condition one or two flashes were presented and the subjects had to indicate how many flashes they perceived. In the A-condition one or two auditory beeps were presented and the participants had to indicate how many beeps they perceived. The stimulus onset asynchrony (SOA) of the two stimuli was varied (factor “SOA” with levels “0 ms” (only one stimulus), “50 ms,” “100 ms,” “150 ms,” “200 ms,” “250 ms,” “300 ms,” “500 ms”). The factor “modality” (V, A, AV) was blocked and the block order randomized between participants. Within each block, SOA was randomly permuted. Seventy trials of each unimodal conditions were presented: 35 trials with only one unimodal stimulus (SOA = “0 ms”) and 35 trials with two unimodal stimuli and the remaining SOAs (5 trials for each of the SOAs “50 ms,” “100 ms,” “150 ms,” “200 ms,” “250 ms,” “300 ms,” “500 ms”).

The AV-block comprised three different conditions: the illusion condition (“illusion” with 2 beeps and 1 flash) as well as two control conditions (“control 1”: 1 beep and 1 flash; “control 2”: 2 beeps and 2 flashes). The first flash and auditory beep were always presented at the same time and the SOAs between them and the second stimuli varied between 50, 100, 150, 200, 300, and 500 ms, as illustrated in Figure 1, upper row. Each of the “illusion,” “control 1,” and “control 2” conditions consisted of 35 trials. All 105 trials were randomly presented within the AV-block. Subjects were instructed to ignore the auditory stimuli and indicate how many visual flashes they perceived.

Responses were made via a response pad (LogiLink® Keypad). Participants were asked to emphasize accuracy over speed. The experiment was programmed in Presentation (Neurobehavioral Systems, CA, USA).

### Statistical Analysis

#### SiFi Data

The average proportion of correct responses for each condition was calculated separately for each participant [numbers ranging between 0.0 (no correct responses) and 1.0 (all responses correct)]. These numbers were used as dependent variables. First, an ANOVA with repeated measures on the unimodal conditions was computed with factor “modality” with levels “visual” (V) and “auditory” (A); factor “SOA” with levels “0 ms,” “50 ms,” “100 ms,” “150 ms,” “200 ms,” “250 ms,” “300 ms,” and “500 ms”; as well as factor “group” with levels relapse-remitting MS patients (“RRMS”), progredient MS (“PMS”) and healthy controls (“HC”).

During the next step of the analysis we computed an ANOVA with repeated measures on the bimodal conditions with factor “condition” with levels “illusion,” “control 1,” and “control 2,” factor “SOA” with levels “50 ms,” “100 ms,” “150 ms,” “200 ms,” “250 ms,” “300 ms,” and “500 ms” as well as factor “group” with levels relapse-remitting MS patients (“RRMS”), progredient MS (“PMS”), and healthy controls (“HC”).

To further disentangle the complex three-way interaction in the bimodal ANOVA, non-parametric Mann-Whitney-U-Tests were computed for comparisons of interest (e.g., comparing “RRMS vs. HC” or “PMS vs. HC” separately for SOAs 200 to 500 ms, s. below). The Bonferroni correction for multiple comparisons was applied.

#### Neuropsychological Tests

We concentrated on several cognitive processes which are usually affected in MS patients (3): information processing ability, speed and flexibility (measured by SDMT, PASAT, TMT-A and TMT B/A); learning capacity (measured by the total number of correctly recalled items in trials 1 to 5 of the VLMT [VLMT total]); memory loss due to forgetting over time (indicated by the “trial 7”–“trial 5” difference in the VLMT [VLMT 5–7]); visuospatial recall memory (measured by the Immediate Recall RCFT trial, RCFT_IR); phonemic and semantic verbal fluency {phonemic and semantic subtests of the RWT [RWTp and RWTs]}.

Premorbid intelligence and cognitive reserve were evaluated and included as covariates by means of calculating patients' years of education and vocabulary (WST-Z-scores) as suggested by Sumowski et al. (24). Depression was measured with BDI and included as a covariate, too. Task-related changes in fatigue were compared between the groups with a univariate ANOVA with “group” as a fixed factor and the relative VAS score as a dependent variable. The relative VAS score indicates how much the fatigue has increased during the neuropsychological testing relatively to the individual baseline score and was computed following the formula (VAS 2-VAS 1)/VAS 1.

For each of the neuropsychological variables a single univariate ANOVA was computed using the direct scores from the tests and with “group” (“RRMS,” “PMS,” “HC”) as a fixed factor and “age,” “years of education,” “WST-Z-Score,” and “BDI” as covariates, thus ensuring that we control for the confounding influence of premorbid intelligence, cognitive reserve, and depression on the neuropsychological performance. The threshold for statistical significance for these ANOVAs was corrected after considering multiple comparisons using the Bonferroni correction and set accordingly to *p* < 0.005.

To test whether cognitive performance of MS patients can be predicted by their multisensory perceptual patterns, we analyzed the data of MS subjects only. Their test results were converted to z-scores. These z-scores were calculated from normalized and normative values existing for each neuropsychological test and then used to compute a stepwise linear regression. The individual number of failed tests of each subject (i.e., tests with below average performance) as an indicator for global neuropsychological impairment was used as a dependent variable, while age, years of education, WST-Z-Score, BDI, the relative VAS-score and proportion of correct responses for the two SiFi illusion conditions with the longest SOA, namely 300 and 500 ms, were used as predictors, since the ANOVA analyses showed that these two illusion conditions differentiate between subgroups (s. below). The threshold for neuropsychological test failure was defined as one or more standard deviations below the reference mean.

To corroborate our findings, we conducted a complementary analysis using a global z-score of the broad neuropsychological test battery based on the procedure described in (25). First, domain-specific z-scores (Z-memory; Z-attention/executive; Z-fluency) were built according to the following formulae, where the different subtests were weighted by their z-score to balance the tasks: Z-memory = (“RCFT_IR” z-score + “VLMT total” z-score + “VLMT 5-7” z-score)/3; Z-attention/executive = (“SDMT” z-score + “PASAT” z-score + “TMT-A” z-score + “TMT B/A” z-score)/4; Z-fluency = (“RWTp” z-score + “RWTs” z-score)/2. The global z-score was obtained by calculating the mean of the z-scores from the three cognitive domains and used as a dependent variable in a linear regression employing the entry method and the illusion condition with the longest SOA 500 ms (since the previous analysis step showed that SOA 500 ms is a significant predictor for the number of failed neuropsychological tests, see Results) as well as the clinical variables age, years of education, WST-Z-Score (indicator for vocabulary and cognitive reserve), BDI (indicative of depression), the relative VAS-score (indicative of task-related fatigue) and the individual disease duration in years.

## Results

### Demographic Parameters

Groups did not differ significantly with regard to the female/male subjects' ratio (chi-square test, *p* > 0.05). The only significant group difference for the visual acuity was for the left eye between HC and PMS (HC > PMS, ANOVA *post-hoc t*-test, *p* = 0.006). However, visual acuity of MS patients did not correlate with their performance in the relevant illusion conditions (SOA 300 and 500 ms), nor with the number of neuropsychological tests with below average performance or the patient's global z-score in the neuropsychological test battery (*p* > 0.05 for all correlations for the left and the right eye).

### ANOVA Unimodal Conditions

The repeated measures ANOVA employing the unimodal conditions demonstrated significant main effects of the factors “modality” and “SOA” as well as a significant interaction between them (Table 2). The close inspection of these results showed that subjects gave more correct responses in the auditory conditions across all SOAs and subgroups (main effect of the factor “modality”). Higher SOAs were associated with an increased number of correct responses (main effect of the factor “SOA”). This effect occurred more quickly for the auditory conditions with average correct responses reaching one of their highest values already at SOA 150 ms (interaction “modality x SOA”). The main effect of the factor “group” was not significant. Moreover, there was no significant interaction of any within-subjects factor with the between-subjects factor “group.”

**Table 2:** Results from the unimodal repeated measures ANOVA with factors “modality” (levels “visual” and “auditory”), “SOA” (levels “0 ms,” “50 ms,” “100 ms,” “150 ms,” “200 ms,” “250 ms,” “300 ms,” and “500 ms”) as well as “group” (levels “RRMS,” “PMS,” “HC”). The Huynh-Feldt-correction for violation of sphericity was applied.

**Main factor/interaction**	**Type III sum of squares**	***F*-value**	**Significance**
Modality	1.613	16.436	<0.001
SOA	49.797	190.400	<0.001
Group	0.158	0.441	n.s.
Modality × group	0.055	0.283	n.s.
SOA × group	0.198	0.379	n.s.
Modality × SOA	5.149	23.516	<0.001
modality × SOA × group	0.399	0.911	n.s.

*“n. s.,” not significant (p > 0.05)*.

### ANOVA Bimodal Conditions

**Figure 2:**
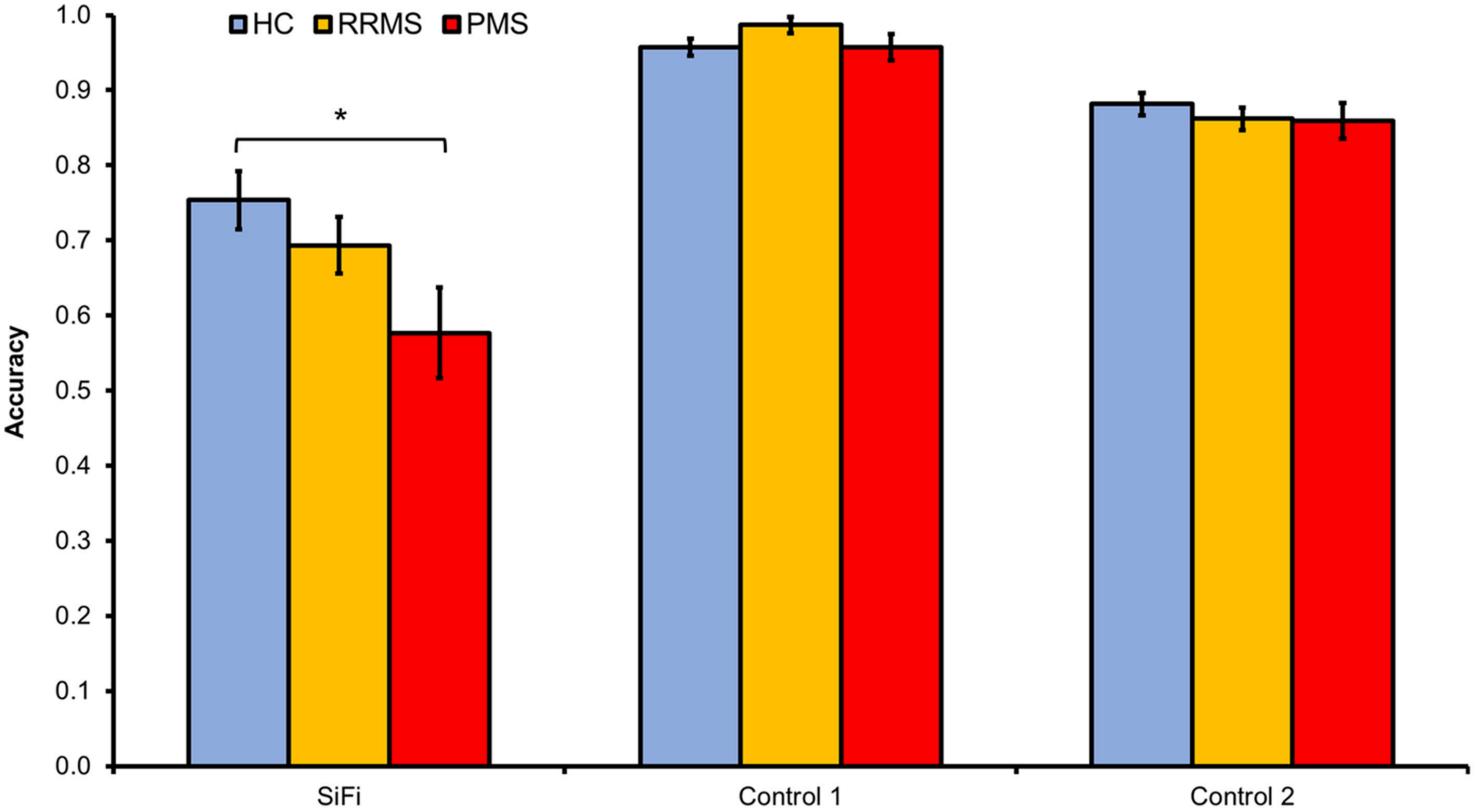
MS patients' responses were less accurate as compared to those of HC in the illusion condition, i.e., MS reported more frequently than HC a second, non-existing visual flash in the SiFi condition, whereas there were no group differences in the non-illusion control conditions (ANOVA interaction “condition × group,” *p* < 0.05). *post-hoc t*-tests revealed that this interaction was driven mainly by the poorer accuracy of PMS as compared to HC in the SiFi condition (average proportion of correct responses for PMS = 0.56 vs. *HC* = 0.75, *p* < 0.05, Bonferroni correction for multiple comparisons). Error bars represent standard error of the mean.

The repeated measures ANOVA using the bimodal conditions demonstrated significant main effects of the within-subjects factors “condition” and “SOA” (Table 3). The highest accuracy was achieved in the condition “control 1,” followed by “control 2” and “illusion” (main effect of the factor “condition”, *post-hoc t*-tests *p* < 0.05). Higher SOAs were associated with an increased number of correct responses (main effect of the factor “SOA,” *post-hoc t*-tests *p* < 0.05). Most importantly, there was a significant interaction “condition × group” (*p* < 0.05). To further disentangle this interaction, we performed *post-hoc t*-tests comparing PMS and RRMS with HC in the illusion as well as the two control conditions. PMS patients achieved far fewer correct responses in the “illusion” condition as compared to HC (average proportion of correct responses for PMS = 0.56 vs. 0.75 for HC, *p* < 0.05, Bonferroni correction for multiple comparisons) while there were no other significant group differences (Figure 2). The main effect of the between-subjects factor “group” was not significant.

**Table 3:** Results from the bimodal repeated measures ANOVA with factors “condition” (levels “illusion,” “control 1,” and “control 2,”) “SOA” (levels “50 ms,” “100 ms,” “150 ms,” “200 ms,” “250 ms,” “300 ms,” and “500 ms”) as well as “group” (levels “RRMS,” “PMS,” “HC”). The Huynh-Feldt-correction for violation of sphericity was applied.

**Main factor/interaction**	**Type III sum of squares**	***F*-value**	**Significance (*p*-value)**
Condition	24.494	86.416	<0.001
SOA	16.530	107.444	<0.001
Group	1.093	2.602	n.s.
condition × group	1.593	2.810	0.049
SOA × group	0.254	0.824	n.s.
condition × SOA	18.317	50.582	<0.001
condition × SOA × group	0.834	1.151	n.s.

*“n. s.,” not significant (p > 0.05)*.

**Figure 3:**
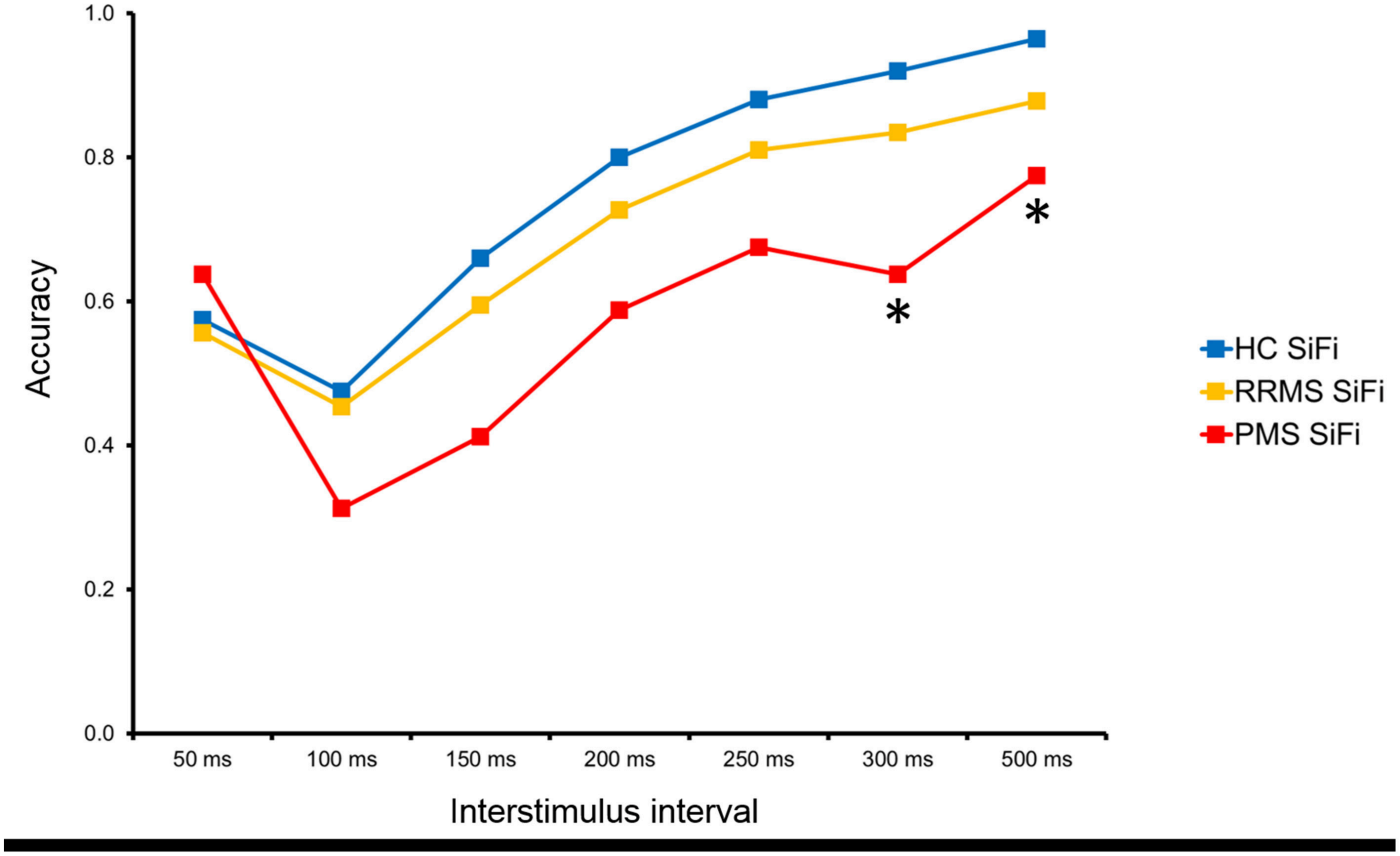
The analysis of the separate interstimulus intervals revealed that PMS continued to perceive the SiFi more often than HC even at interstimulus time intervals of 300 and 500 ms (* = *p* < 0.05, Bonferroni corrected).

The three-way ANOVA interaction “condition × SOA × group” did not reach significance. The close inspection of this complex interaction hinted, however, at possible group differences in the illusion condition at longer SOAs. Based on previous work demonstrating that healthy subjects stop perceiving the illusion at SOAs longer than 150 ms, but patients with mild cognitive impairment continue perceiving it even at longer SOAs (13), we compared the three groups separately using Mann–Whitney U tests on the SOAs of 200, 250, 300, and 500 ms in the “illusion” condition only. PMS subjects had a significantly lower average proportion of correct responses than healthy controls for all SOAs in the “illusion” condition. After applying a correction for multiple comparisons, the group differences for SOAs of 300 and 500 ms remained significant (*p* < 0.05, Bonferroni correction for multiple comparisons) (Figure 3).

### Neuropsychological Test Performance

Groups did not differ significantly on their relative VAS score (*p* > 0.05), indicating that fatigue did not increase group-dependently during the neuropsychological testing. The single univariate ANOVAs demonstrated at first significant group differences for RCFT_IR (HC > RRMS), SDMT (HC > RRMS > PMS), VLMT total (HC > RRMS, HC > PMS) and RWTp (HC > RRMS, HC > PMS) after controlling for age, years of education, vocabulary, and depression. However, after applying the Bonferroni correction for multiple comparisons only the group difference in SDMT remained significant (HC > RRMS > PMS, Type III sum of squares = 1327.001, *F*-value = 7.728, *p* < 0.005). The mean number of failed tests for the MS patients was 3.1 (min 0, max 8, SD 2.1). RRMS and PMS did not differ significantly on their number of failed tests (*p* > 0.05).

### Cognitive Impairment and SiFi

The stepwise linear regression modeling the relationship between the individual number of failed tests as dependent variable and age, years of education, WST-z-score, BDI, the relative VAS-score and proportion of correct responses for the two SiFi illusion conditions SOA 300 and 500 ms revealed that only the average proportion of correct responses for the SOA 500 ms in the illusion condition contributed significantly to explaining the variance of the dependent variable “number of failed tests” in MS patients (*t* = −2.826, partial correlation = −0.374, *p* = 0.007, see Table 4, Supplementary Figure 1).

**Table 4:** Results from the stepwise linear regression (model “1”) testing the relationship between the individual number of failed tests as dependent variable and age, years of education, WST-z-score, BDI, the relative VAS-score and proportion of correct responses for the two SiFi illusion conditions SOA 300 and 500 ms as independent variables.

**Model**	**R**	**R square**	**F change**	**Significance**	
1	0.374	0.140	7.988	***0.007***	
**Variable**	**B (unstandardized)**	**Std. error**	**t**	**Significance**	**Partial correlations**
Constant	5.076	0.826	6.143	<0.001	
Illusion SOA 500 ms	−2.577	0.912	−2.826	***0.007***	−0.374
Age			−1.770	0.083	−0.247
Years of education			−1.869	0.068	−0.261
BDI score			−0.836	0.407	−0.120
WST-z score			−0.347	0.730	−0.050
VAS relative score			−0.626	0.534	−0.090
Illusion SOA 300 ms			−0.172	0.864	−0.025

The second linear regression tested a model where the global z-score was used as a dependent variable and the illusion condition with the longest SOA 500 ms as well as age, years of education, WST-Z-score, BDI, the relative VAS-score and the individual disease duration were employed as independent variables. It revealed a significant result for the model (*R* = 0.572, *R* square 0.328, *p* = 0.012), whereby only the average proportion of correct responses for the SOA 500 ms in the illusion condition (*t* = 2.163, *p* = 0.036) and years of education (*t* = 2.028, *p* = 0.049) contributed significantly to explaining the variance of the dependent variable “global z-score” in MS patients, thus corroborating the results from the first analysis (see Table 5 for details and Supplementary Figure 1).

**Table 5:** Results from the entry linear regression model (model “2”) testing the relationship between the global z-score as dependent variable and the SiFi condition SOA 500 ms as well as age, years of education, WST-Z-score, BDI, the relative VAS-score and the disease duration as independent variables.

**Model**	**R**	**R-square**	**F-change**	**Significance**	
2	0.572	0.328	2.994	***0.012***	
**Variable**	**B (unstandardized)**	**Std. error**	**t**	**Significance**	**Partial correlations**
Constant	−3.824	1.222	−3.129	0.003	
Illusion SOA 500 ms	0.527	0.244	2.163	***0.036***	0.313
Age	0.011	0.006	1.970	0.055	0.288
Years of education	0.179	0.088	2.028	***0.049***	0.295
BDI score	0.009	0.008	1.157	0.254	0.174
WST-z score	0.052	0.107	0.486	0.630	0.074
VAS relative score	−0.096	0.123	−0.779	0.440	−0.118
Disease duration	−0.010	0.009	−1.136	0.262	−0.171

## Discussion

MS patients performed worse than healthy control subjects in SDMT indicating worse general information processing ability and information processing speed. Within the MS group, performance of PMS patients was significantly lower than that of RRMS patients. These results are compatible with the findings reported in the literature (3).

Furthermore, perception of the SiFi differed across MS and HC groups. In particular, MS patients seemed to be more susceptible to the multisensory illusion than healthy control subjects. These findings resemble the results of Chan et al. (13), where patients with mild cognitive impairment (MCI) perceived the illusion more often than controls. Interestingly, MCI patients perceived the illusion for SOAs of up to 300 ms, while we demonstrated that PMS patients perceive the SiFi at even longer SOAs of 500 ms.

Essentially, increased susceptibility to SiFi was strongly correlated with the number of failed neuropsychological tests and the global z-score of the neuropsychological test battery used in MS patients. In the computed linear regressions, the perception of the illusion at the longest SOA of 500 ms contributed significantly to explaining the variance of the global cognitive impairment. Remarkably, MS patients who perceived the multisensory illusion at an SOA that was more than three times longer than the SOA at which the illusion was disrupted for healthy controls exhibited the most pronounced cognitive deficits.

One possible explanation for the fact that aberrant multisensory perception in MS patients predicted for cognitive deficits is a dysfunction of early cortical processes mainly involved in unimodal perception. Indeed, Shams et al. (26) demonstrated a sound-associated modulation of visually induced MEG activity in occipital and parietal scalp locations of healthy subjects as early as 35–65 ms from the onset of the visual stimulus. Furthermore, de Haas et al. (6) found that individual differences in proneness to the illusion in healthy subjects were strongly correlated with local gray matter volume in early retinotopic visual cortex. Participants with smaller early visual cortices were more prone to the illusion. Thus, it is possible that neuronal damage to early stages of the visual and/or auditory pathways of MS patients results in increased proneness to the illusion. This, however, would not explain the cognitive deficits. More importantly, dysfunctional unimodal visual and auditory systems —e.g., due to demyelination in MS—which are significant enough to disrupt normal multisensory integration should have resulted in worse performance in our unimodal experimental trials, too. However, MS patients did not perform worse than HC in unimodal or bimodal control tasks. Moreover, Michail and Keil have recently shown that high cognitive load increases the susceptibility for the illusion in healthy subjects which hints at top-down cognitive influence on multisensory integration (8).

Therefore, we suggest that dysfunctional multisensory perception and cognitive deficits in MS share another common cause: impaired brain connectivity due to neuronal damage. In their original study Shams et al. demonstrated that illusion-associated MEG activity was also modulated in the occipital and parietal areas as well as anterior areas at a later (approximately 150 ms post-stimulus) onset (26). Furthermore, using MEG Keil et al. showed that sound-induced visual illusory perceptions were preceded by alpha and beta-band phase synchrony changes between several cortical areas (visual and auditory cortices, parietal and frontal cortical areas) (7) and beta-band phase synchrony is known to play an important role in the large-scale synchronization of functionally specialized brain regions (21). Similarly, Balz et al. employed MR spectroscopy and electroencephalography and found robust relationships between GABA concentration, gamma band oscillations and the SIFI perception rate in healthy subjects and suggested that the GABA level shapes individual differences in audiovisual perception through its modulating influence on gamma band oscillations (5). In an event-related potentials (ERP) study, Mishra et al demonstrated an early modulation of visual cortex activity at 30–60 ms after the second sound, which was larger in amplitude in subjects who saw the illusory flash more frequently (27). Further analysis found that short-latency ERP activity localized to auditory cortex and polymodal cortex of the temporal lobe and associated with gamma bursts in visual cortex determines the perception of the illusion. This suggests that the second sound triggers an interplay between auditory and visual cortical areas and results in perception of the illusory second flash (27). Thus, interactions between cortical areas seem to be crucial for viable multisensory perception.

Two mechanisms of MS-related dysfunction of these interactions leading to concomitant cognitive deficits are possible. First, predominantly white matter-related demyelinating and in the course of disease also axonal damage could impair the pathways connecting different brain areas, resulting in an insufficient interplay of the associative cortices, thus compromising crossmodal but also cognitive processes. Indeed, brain imaging studies have reported altered functional as well as structural connectivity in MS related to sensorimotor as well as cognitive symptoms (28–30). Second, direct damage to associative areas due to cortical lesions may impair their integrative function, thus disrupting both cognitive and multisensory processes. In the light of the recent development of MRI brain imaging it has become easier to visualize cortical lesions. Cortical lesions have been recently recognized as characteristic of multiple sclerosis, contributing to the MRI criteria for dissemination in space (31). Furthermore, extensive cortical damage at the onset of the disease is associated with florid inflammatory clinical activity and predisposes to a rapid occurrence of the progressive phase of MS (32). Moreover, cortical lesions are associated with cognitive and physical disability in MS (33, 34). Thus, MS-related demyelinating of white matter tracts which support the interplay between cortical regions in the healthy brain as well as cortical MS lesions might be the common underpinning of the observed findings: once the common neural pathways are disrupted, both crossmodal information transfer and cognitive processing are diminished.

An interesting question is how our findings relate to other works showing aberrant SiFi perception in neuropsychiatric disorders such as autism and schizophrenia. In autism, one study using the SiFi found a wider temporal binding window in autism spectrum disorder compared to controls (35), which implies a higher susceptibility for the illusion, similar to our findings in MS patients, while another study reported a narrower temporal binding window and thus a diminished susceptibility for the illusion in autism patients (36). Similarly, reduced perception of the SiFi has been reported for schizophrenia patients (37). An interesting observation has been made also by Brighina et al. who demonstrated that compared with controls, migraine patients are less prone to perceive the sound-induced illusion, especially during migraine attacks and/or if they had a migraine with an aura (38), which argues that a state of cortical hyperexcitability diminishes the effect of the illusion. Finally, Chan et al. showed that patients with MCI perceive the illusion more often than controls (13). These findings from clinical populations suggest that brain disorders characterized by an aberrant neural connectivity, such as autism and schizophrenia, exhibit also an altered SiFi perceptual pattern and that cognitive impairment due to neurodegenerative processes such as those seen in MCI might share a common neural basis with the SiFi.

Our findings not only show for the first time that multisensory perception in MS might be impaired but also imply that SiFi performance reflect cognitive deficits. We do not suggest that it should substitute SDMT, PASAT or other screening measures, as numerous studies have provided strong evidence for the utility of SDMT and PASAT in screening for cognitive deficits (3). Instead, we propose that SiFi can be employed complementary or alternatively in certain situations. Using SiFi in the clinical practice has several advantages. While the task takes approximately not more than 10 min, SiFi performance correlates with the global neuropsychological impairment, as indicated by the association between the susceptibility for the illusion and the performance in a broad neuropsychological battery measuring various cognitive deficits, e.g., information processing ability and speed, learning capacity, phonemic, and semantic verbal fluency, etc. The SiFi task is easy to use and does not require any special equipment apart from a laptop or mobile device and headphones. There are no additional costs and no extensive training for the staff is required. Furthermore, it is language- and education-independent. Importantly, there are no learning effects on the SiFi illusion which makes it particularly feasible in monitoring cognitively impaired patients or screening more often for neuropsychological deficits in patients with active disease (10, 11).

Obviously, SiFi cannot replace neuropsychological tests. However, it offers an opportunity for neurologists—especially in outpatient care—to screen their MS patients for cognitive deficits with minimal time and resource investment. Patients exhibiting aberrant illusion perception (i.e., perceiving the SiFi at an SOA of 500 ms) could be referred to a more extensive neuropsychological investigation. Importantly, since SiFi is a multisensory illusion without significant learning effects, it can be applied multiple times during the course of disease, in particular for monitoring progression of cognitive deficits over time. According to our data, patients with progressive MS are particularly affected by the increased susceptibility to SiFi.

One limitation of the current study is the sample size. This, as well as the rather conservative analyses, i.e., controlling for multiple covariates in the ANOVAs and using the rigorous Bonferroni method, may have contributed to the fact that when directly comparing MS patients and HC in the neuropsychological testing and correcting for multiple comparisons, group differences remained significant only for the SDMT. Certainly, studies with larger patient samples are necessary before introducing SiFi to the clinical practice. Further aspects of MS-treatment can be additionally investigated in future studies with more patients: e.g., monitoring cognitive function under a particular disease-modifying therapy by using SiFi or monitoring rehabilitation for cognitive dysfunction.

Another possible limitation of the paradigm is the visual impairment typically seen in MS patients. One could argue that this would possibly confound the illusion measurements. However, in our study, the only significant group difference for the visual acuity was between the PMS patients and the HC for the left eye. Furthermore, there was no significant group difference in the unimodal (i.e., only visual or only auditory stimulation) control conditions and there was no significant correlation between the performance in the illusion conditions and the visual acuity. Last but not least, we believe that due to the nature of the visual stimulation (e.g., a very simple light flash on the computer screen from a reading distance) even MS patients with some visual impairment would not find it difficult to detect the stimulus.

An interesting observation was that the variable “years of education” was a significant predictor for the global neuropsychological performance in MS patients besides the illusion performance, as seen in the second regression model. This corresponds with other studies which reported a relationship between the highest degree of education and the cognitive performance of MS patients, suggesting that the formal education can exert a positive, possibly protective influence over neuropsychological functions, serving as a “cognitive reserve” (3). Future studies testing for the feasibility of the sound-induced flash illusion as a screening for global neuropsychological impairment should take these results into account.

Cognitive decline is recognized as a prevalent and devastating symptom of MS (3). To our best knowledge, our study is the first to show that MS patients exhibit altered multisensory perception in the SiFi task and that their susceptibility to the perceptual illusion is correlated with their number of failed neuropsychological tests. Thus, SiFi can be considered for further research as a screening test for global cognitive impairment in MS patients.

## Ethics Statement

This study was carried out in accordance with the recommendations of the ethics committee of the University of Frankfurt Medical Faculty with written informed consent from all subjects. All subjects gave written informed consent in accordance with the Declaration of Helsinki. The protocol was approved by the ethics committee of the University of Frankfurt Medical Faculty.

## Author Contributions

YY: design and conceptualized study, analyzed the data, drafted the manuscript for intellectual content, Interpreted the data, revised the manuscript for intellectual content; HB, DS: analyzed the data, interpreted the data, major role in the acquisition of data; CB, LF: analyzed the data, major role in the acquisition of data; MN, JK: design and conceptualized study, revised the manuscript for intellectual content; SF, MB, JG: design and conceptualized study, interpreted the data, revised the manuscript for intellectual content; CF: interpreted the data, revised the manuscript for intellectual content.

### Conflict of Interest Statement

YY has been supported by travel grants from Novartis and Sanofi Genzyme, has received an honorarium for active participation in an advisory board by Sanofi Genzyme as well as a speaking honorarium by Roche. No financial support has been received for this particular project. The remaining authors declare that the research was conducted in the absence of any commercial or financial relationships that could be construed as a potential conflict of interest.

## Supplementary Material

Supplementary Figure 1 The binned scatter plots for the independent variable “SiFi SOA 500 ms” and the dependent variables “number of failed tests” and “z global score” from the two regression models are shown in the upper row. The lower row of scatter plots demonstrates the partial regression plots for the two significant predictors “years of education” and “SiFi SOA 500 ms” from the second regression model after controlling for the influence of the other variables.
