# Supplementary figures and images for: Cognitive Impairment in Multiple Sclerosis Is Reflected by Increased Susceptibility to the Sound-Induced Flash Illusion
Source: Front Neurol. 2019 Apr 12;10:373. doi: 10.3389/fneur.2019.00373 (PMC6474182; doi:10.3389/fneur.2019.00373)

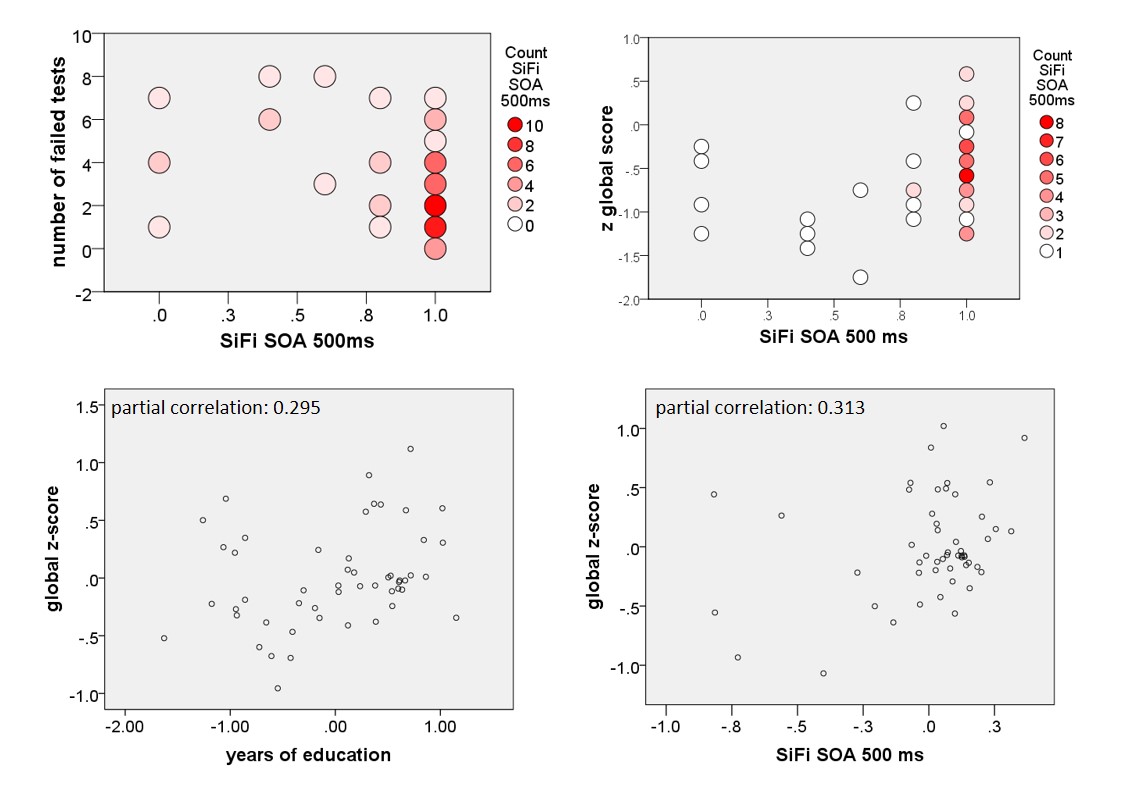

Supplement: Supplementary Figure 1 — The binned scatter plots for the independent variable “SiFi SOA 500 ms” and the dependent variables “number of failed tests” and “z global score” from the two regression models are shown in the upper row. The lower row of scatter plots demonstrates the partial regression plots for the two significant predictors “years of education” and “SiFi SOA 500 ms” from the second regression model after controlling for the influence of the other variables. [file Image_1.JPEG]
